# Induced Pluripotent Stem Cells Derived from a CLN5 Patient Manifest Phenotypic Characteristics of Neuronal Ceroid Lipofuscinoses

**DOI:** 10.3390/ijms18050955

**Published:** 2017-05-03

**Authors:** Kristiina Uusi-Rauva, Tea Blom, Carina von Schantz-Fant, Tomas Blom, Anu Jalanko, Aija Kyttälä

**Affiliations:** 1 National Institute for Health and Welfare, Genomics and Biomarkers Unit, P.O. Box 104, 00251 Helsinki, Finland; kristiina.uusi-rauva@helsinki.fi (K.U.-R.); tea.blom@gmail.com (T.B.); anu.jalanko@thl.fi (A.J.); 2 Folkhälsan Institute of Genetics, P.O. Box 63, University of Helsinki, 00014 Helsinki, Finland; 3 Institute for Molecular Medicine Finland, FIMM, Tukholmankatu 8, 00290 Helsinki, Finland; cvonschantzfant@gmail.com; 4 Department of Anatomy, Faculty of Medicine, University of Helsinki, Haartmaninkatu 8, 00290 Helsinki, Finland; tomas.blom@helsinki.fi

**Keywords:** CLN5, iPS cells, NCL, sphingolipid, lysosomal storage disease

## Abstract

Neuronal ceroid lipofuscinoses (NCLs) are autosomal recessive progressive encephalopathies caused by mutations in at least 14 different genes. Despite extensive studies performed in different NCL animal models, the molecular mechanisms underlying neurodegeneration in NCLs remain poorly understood. To model NCL in human cells, we generated induced pluripotent stem cells (iPSCs) by reprogramming skin fibroblasts from a patient with CLN5 (ceroid lipofuscinosis, neuronal, 5) disease, the late infantile variant form of NCL. These CLN5 patient-derived iPSCs (CLN5Y392X iPSCs) harbouring the most common *CLN5* mutation, c.1175_1176delAT (p.Tyr392X), were further differentiated into neural lineage cells, the most affected cell type in NCLs. The CLN5Y392X iPSC-derived neural lineage cells showed accumulation of autofluorescent storage material and subunit C of the mitochondrial ATP synthase, both representing the hallmarks of many forms of NCLs, including CLN5 disease. In addition, we detected abnormalities in the intracellular organelles and aberrations in neuronal sphingolipid transportation, verifying the previous findings obtained from *Cln5*-deficient mouse macrophages. Therefore, patient-derived iPSCs provide a suitable model to study the mechanisms of NCL diseases.

## 1. Introduction

Neuronal ceroid lipofuscinoses (NCLs) are inherited lysosomal storage diseases and constitute the most common group of pediatric neurodegenerative disorders. NCLs are caused by mutations in at least 14 different genes (*CLN1*–*CLN14*) and share several clinical and pathological features including visual impairment, seizures, brain atrophy and storage of lysosomal autofluorescent ceroid, lipofuscin-like material [1,2,3,4]. The age of NCL onset is variable [1,4]. To date, there are no effective therapies for any form of NCL, and these disorders are invariably fatal.

Here, we focus on CLN5 (ceroid lipofuscinosis, neuronal, 5) disease, a late infantile variant NCL (MIM#256731), caused by mutations in the *CLN5* gene [5,6,7]. Most pathogenic *CLN5* mutations result in a late infantile variant phenotype, but atypical phenotypes have also been identified, including those possibly modified by mutations in other genes [1,4,8,9]. In the late infantile variant form of the CLN5 disease, the first symptoms, motor clumsiness and attention disturbances, appear between 4–7 years of age and are followed by progressive visual failure, motor and mental decline, ataxia, myoclonia and epilepsy, and an early death between the second and fourth decades of life [7,10]. The CLN5 patients’ brains show the earliest and most severe atrophy in the cerebellum accompanied by storage deposition, destruction of cerebral neurons, astrocytosis and hypomyelination [11,12,13].

We previously generated a *Cln5* knock-out (ko) mouse model [14] which presents a relatively late onset neurodegenerative disease phenotype with visual/cognitive dysfunction, progressive accumulation of autofluorescent storage material, loss of GABAergic interneurons, synaptic pathology, and a marked glial activation and hypomyelination preceding neuronal loss, mostly pronounced in the thalamocortical system [14,15,16]. In addition, *Cln5* ko mice suffer from several neurological defects, including mild motor dysfunction, and exhibit progressive weight loss and brain atrophy [14].

The CLN5 protein is a soluble lysosomal glycoprotein [17,18] that is expressed ubiquitously, including in neurons and glia [15,19]. The function of the CLN5 protein is currently unknown. Gene expression profiling of the *Cln5* ko mouse brain revealed several affected pathways [14,20]. In vitro protein interaction studies suggest that different NCL proteins, including CLN5, participate in shared protein interaction networks/functional pathways [21,22,23,24]. We have previously shown that CLN1 and CLN5 share a common interaction partner, the F1 subunit of the ATP synthase [24,25], a well-known mitochondrial protein, also implicated in the function of cholesterol transport across the plasma membrane [26]. The involvement of both CLN5 and CLN1 in cholesterol metabolism is also suggested by observed abnormalities in the cellular and systemic lipid metabolism in the corresponding mouse models [15,25,27]. Furthermore, *Cln5* ko mice exhibit disturbances in intracellular sphingolipid transport, studied using a fluorescent sphingolipid analogue, BODIPY FL C5-lactosylceramide [15]. Defects in sphingolipid metabolism have also been detected in CLN5 patients’ fibroblasts, observed as decreased synthesis of the sphingolipid species ceramide and ceramide-derived lipids [28]. CLN5 has also been implicated to function in Rab7-mediated endosomal sorting [29], similar to another NCL protein, CLN3 [30].

Several other vertebrate models have also been used to study disease mechanisms of CLN5 and other forms of NCLs [31,32]. The problem, however, with established CLN5 animal models is that they may not fully recapitulate the full spectrum of phenomena associated with human disease. Another obstacle in modelling NCL diseases is that human neurons, the main disease-affected cell type, are not easily available. This limitation can be overcome by using reprogrammed patient-derived cells, induced pluripotent stem cells (iPSCs), and differentiating them into neural lineage cells. Thus, iPSCs provide not only an unlimited source of patient-derived cells for disease modelling but can also be used for drug screening applications, and perhaps in the future, in regenerative medicine. Our results suggest that the iPSC model for CLN5 disease recapitulates the phenotypic characteristics of the human disease and may be a helpful tool for understanding the molecular mechanisms of the CLN5 disease and NCLs in general.

## 2. Results

### 2.1. Generation and Characterisation of CLN5Y392X iPS Cells

**Figure 1:**
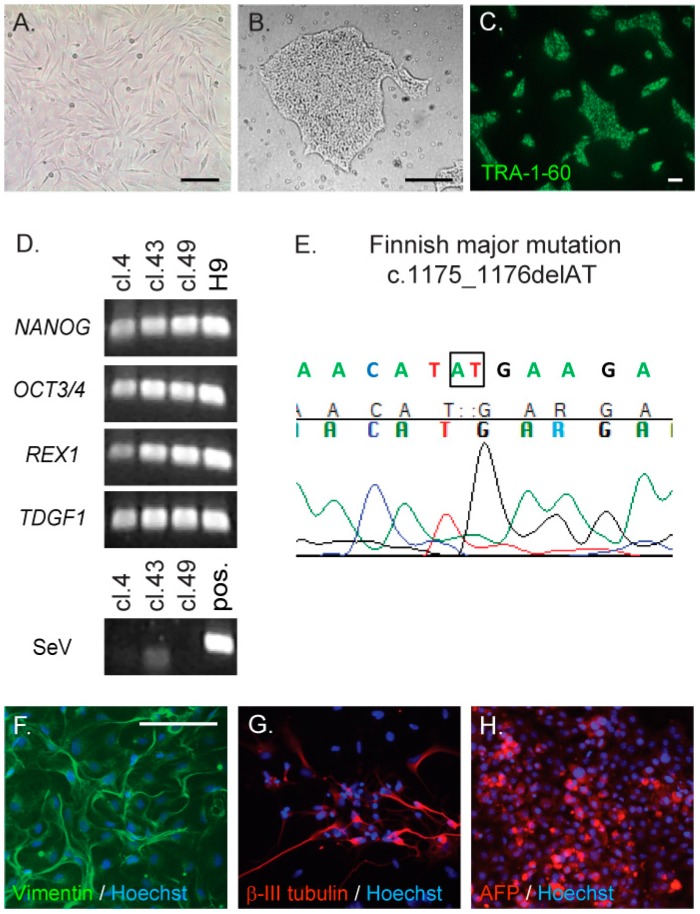
Generation and characterisation of iPS cell lines derived from the CLN5 (ceroid lipofuscinosis, neuronal, 5) patient’s fibroblasts. The CLN5 patient’s fibroblasts (**A**) were reprogrammed to induce pluripotent stem cells (iPSCs) using a Sendai virus-mediated delivery of the four Yamanaka factors. CLN5Y392X iPSC clones were collected on the basis of colony morphology ((**B**) a representative image shown); and the colonies were stained for TRA-1-60, a specific stem cell marker ((**C**) shown for one representative clone); RT-PCR was used to detect the expression of endogenous stem cell marker genes, *NANOG*, *OCT3/4*, *REX1* and *TDGF1*, and the loss of virus expression confirmed by using primers specific for Sendai virus (SeV) ((**D)** shown for the three characterised CLN5Y392X iPSC clones (cl.) and a positive control hES cell line, H9. Sendai virus-infected fibroblasts were used as a positive control (pos.) in analysis of Sendai viral expression); The presence of the original CLN5 disease causing mutation in each analysed CLN5Y392X iPSC clone was confirmed by sequencing ((**E**) illustrated from one representative clone, nucleotides in the box represent second and third nucleotides of the codon TAT, and are missing in mutated DNA); Pluripotency of the three characterised CLN5Y392X iPSC lines was verified by their ability to differentiate into the three germ layers, mesoderm, ectoderm and endoderm, detected by staining with vimentin (**F**), β-III tubulin (**G**) and α-fetoprotein (AFP, (**H**)) antibodies, respectively (shown for one representative clone with nuclear staining shown in blue); Bar 100 µm (**A**,**F**–**H**), 300 µm (**B**) and 50 µm (**C**).

Fibroblasts from the CLN5 patient (Figure 1A) were reprogrammed by the expression of *SOX2*, *OCT3/4*, *KLF4* and *MYC* after infection with a Sendai virus delivery vector, in order to produce integration-free CLN5Y392X iPSCs. Control iPSC lines from healthy donors have been described previously [33,34]. The initial CLN5Y392X iPSC colony selection was based on morphologic resemblance to human embryonic stem cell (ESC) colonies (Figure 1B). Altogether, 10 different CLN5 patient iPSC clones were collected and expanded for further analyses. Three of the expanded clones (clones 4, 43 and 49) were characterised in detail. All three selected CLN5Y392X iPSC clones expressed typical stem cell marker proteins, TRA-1-60, OCT3/4 and SSEA3 (Figure 1C and Figure S1). Expression of human ESC marker genes (*NANOG*, *OCT3/4*, *REX1* and *TDGF1*) in the three selected CLN5Y392X iPSC clones was verified by RT-PCR. The human embryonic stem (ES) cell line H9 was used as a positive control (Figure 1D). All three CLN5Y392X iPSC clones were negative for Sendai virus (SeV) expression, demonstrating the disappearance of the virus and the transgenes (Figure 1D). Sequencing confirmed the presence of the homozygous disease-associated mutation in the *CLN5* gene (c.1175_1176delAT, p.Tyr392X) in all analysed CLN5Y392X iPSC clones (Figure 1E). The three selected CLN5Y392X iPSC clones were also determined to have normal karyotypes (Supplementary Materials and Methods, Figure S2). The pluripotent nature of CLN5Y392X iPSC clones was demonstrated through their ability to form embryoid bodies (EBs) and differentiate into all three germ layers *in vitro*, detected by mesodermal marker vimentin, ectodermal marker β-III tubulin, and endodermal marker α-fetoprotein (AFP) (Figure 1F–H).

### 2.2. CLN5Y392X iPSC Differentiate into Neural Lineage Cells and Show Lysosomal and Endoplasmic Reticulum Abnormalities

To be able to characterise whether CLN5Y392X iPSC-derived cells serve as phenotypic models for the CLN5 disease, we decided to concentrate on the most relevant cellular findings. Most NCL proteins localise to the lysosomes or to the endoplasmic reticulum (ER), and CLN5 has been suggested to mediate several interactions between these proteins [17,24]. In addition, depletion of CLN5 in Hela cells has been suggested to result in fragmentation of the trans-Golgi network (TGN) and lysosomal mislocalisation of cation-independent mannose 6-phosphate (CI-MPR) [29], which both could serve as markers for the disease. However, based on our data (Figures S3–S6), such phenomena were not apparent in the patient’s fibroblasts used in this study for the production of CLN5Y392X iPSCs. The steady-state TGN46 staining was found to be compact in both control and CLN5 patient’s fibroblasts when analysed by immunofluorescence microscopy, and showed no statistically significant difference in the total size of TGN between the control and CLN5-deficient cells when analysed by ScanR automated high-content microscopy followed by quantitative image analysis of TGN46-positive structures (Figure S3 and Supplementary Materials and Methods). Similarly, no difference was found in the steady-state localisation of CI-MPR between the CLN5 and control fibroblasts, even when analysed by automated high-content microscopy (Figure S4). Furthermore, blocking protein synthesis by cycloheximide [35] showed no disappearance of CI-MPR, but instead, a compact TGN-like staining was retained in both cell lines (Figure S4 and Supplementary Materials and Methods). Since patient fibroblasts may have developed compensatory mechanisms, we repeated both the TGN and CI-MPR analyses in healthy control fibroblasts, in which CLN5 was separately depleted with three different RNAi probes (Supplementary Materials and Methods). However, we could still not detect any major differences in the TGN or CI-MPR staining between CLN5-depleted and control siRNA-transfected fibroblasts (Figures S5 and S6). Due to clear discrepancy between the results showing TGN and CI-MPR characteristics in the CLN5 deficiency, we decided to concentrate on the most relevant cell organelles for NCLs, the lysosomes and the ER, and perform the respective analyses in the CLN5Y392X iPSC-derived cells of neural lineage.

**Figure 2:**
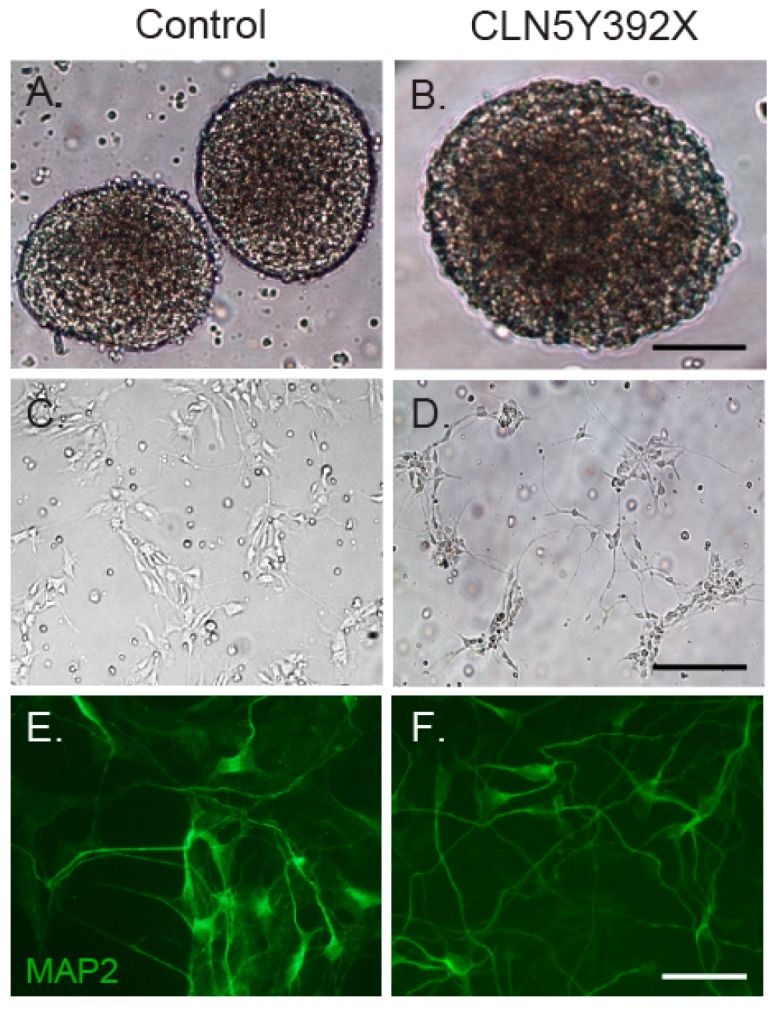
Neural differentiation of control and CLN5Y392X iPSC lines. iPS cells were differentiated into neural progenitor cells (NPCs), which grow as neurospheres in a suspension culture (**A**,**B**); Neurospheres were further differentiated on poly-l-ornithine/laminin-coated plates (**C**,**D**); At day seven, cultures were fixed and stained with the neuron-specific marker microtubule-associated protein 2 (MAP2) (green) (**E**,**F**); Bar 400 µm (**A**,**B**), 200 µm (**C**,**D**) and 50 µm (**E**,**F**).

To do so, we differentiated control and CLN5Y392X iPSC lines at passages 15–23 into neural lineage cells. The differentiation was started with neural induction, followed by expansion of the neural progenitor cells in neurospheres (Figure 2A,B) and further maturation of neurosphere-derived cells in adherent cultures (Figure 2C,D). Both control and CLN5Y392X iPSCs were able to produce cells with neuronal morphology (Figure 2C,D), which expressed a neuron-specific marker microtubule-associated protein 2 (MAP2) already at day seven (Figure 2E,F).

**Figure 3:**
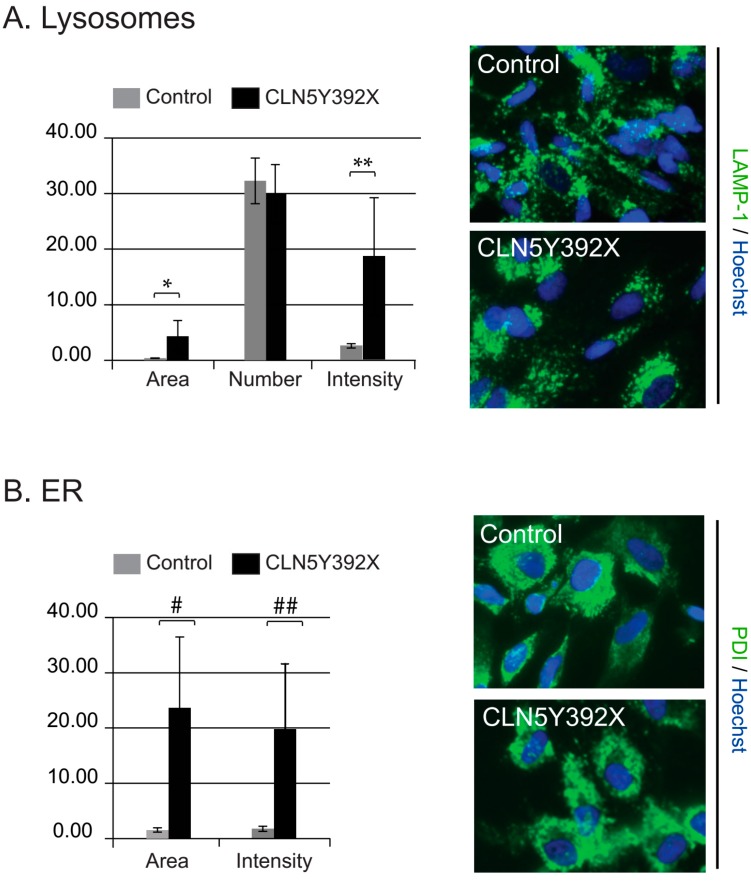
Analyses of lysosomes and the endoplasmic reticulum (ER) in iPSC-derived neural lineage cells. iPSC-derived neurospheres were further differentiated in adherent cultures. After ten days in an adherent culture, the cells were processed for nuclear (Hoechst 33258, blue), and lysosomal (LAMP-1, green) or ER (PDI, green) staining. Representative ScanR microscope images of lysosomal and the ER compartments ((**A**,**B)**, respectively) are shown. Quantitative image analyses show the average values (arbitrary units) of total area, the number and intensity of vesicular LAMP-1-positive compartments in control and CLN5Y392X neural lineage cells (**A**); the size of the ER was determined as the area of PDI-stained structures (**B**). All columns represent the average values of 6–8 replicates each normalised to the number of cells analysed in the replicate (average of 1231 and 1183 cells per replicate for LAMP-1 and ER analysis, respectively). The error bars represent standard deviations (stdevp). *p*-values, 0.0243 (*), 0.0183 (**), 0.0119 (#) and 0.0140 (##) (two-tailed Student’s *t* test).

Next, neurosphere-derived cells differentiated for ten days in adherent culture were fixed and stained with LAMP-1 and PDI antibodies to detect lysosomes and the ER, respectively. High-content microscopy followed by quantitative image analysis showed that both the intensity and the total area of LAMP-1-stained lysosomes were significantly increased in CLN5Y392X iPSC-derived neural lineage cells compared to control cells (Figure 3A). However, the number of lysosomes was relatively uniform in both cell lines (Figure 3A). In addition, the size of the ER and the intensity of PDI staining were clearly increased in CLN5-deficient cells compared to those detected in control cells (Figure 3B).

#### CLN5Y392X iPSC-Derived Neural Lineage Cells Show Accumulation of Storage Material

**Figure 4:**
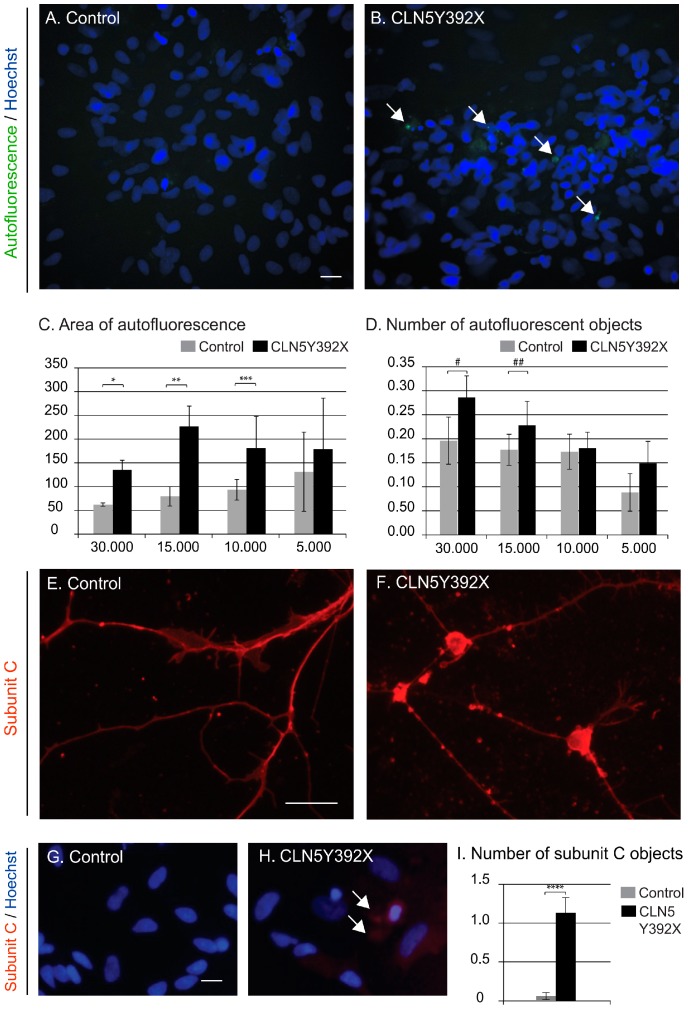
Intracellular storage accumulation in iPSC-derived neural lineage cells. Appearance of storage material within cells analysed by measuring the amount of autofluorescence (**A**–**D**) and subunit C staining (**E**–**I**). Representative images showing autofluorescent compartments (green) and nuclei (Hoechst 33258, blue) in control (**A**) and CLN5Y392XiPSC-derived neural lineage cells (**B**) viewed using a high-content microscope; Quantitative image analysis shows the average area of autofluorescent compartments (**C**) as well as the average number of autofluorescent objects (**D**) in control (grey bars) and CLN5-deficient (black bars) cells (arbitrary units). The *x* axis indicates the plated cell number per 96-well (**C**,**D**). Each column in (**C**,**D**) represents the average value of four replicates per condition each normalised to the number of cells analysed in the replicate (average of 1379 cells per replicate); Representative images of control (**E**) and CLN5Y392XiPSC-derived neural lineage cells (**F**) stained with a polyclonal antibody against subunit C of the mitochondrial ATP synthase (red) and viewed using a traditional epifluorescence microscope; High-content immunofluorescence microscopy images of subunit C staining in control (**G**) and CLN5Y392X neural lineage cells (**H**); Results from the quantitative image analysis showing the average number of subunit C positive objects in control (grey bars) and CLN5-deficient cells (black bars) (**I**). Both columns in (**I**) represent the average value of 2–3 replicates each normalised to the number of cells analysed in the replicate (average of 587 cells per replicate). All error bars in the figure represent standard deviations (stdev). White arrows indicate the presence of autofluorescent/stained storage material. Bar 20 µm. *p*-values, 0.0024 (*), 0.0004 (**), 0.0371 (***), 0.018 (#), 0.0708 (##) and 0.0027 (****) (one-tailed Student’s *t* test).

CLN5 patients’ cells accumulate intracellular autofluorescent lipofuscin-like material, containing subunit C of mitochondrial ATP synthase as the major storage component. To examine the presence of storage material in CLN5Y392X iPSC-derived neural lineage cells, control and CLN5-deficient cells were differentiated for 14 days, and then microscopically analysed for autofluorescence and stained subunit C accumulation. The automated quantitative microscopy analyses of CLN5Y392X iPSC-derived neural lineage cultures showed an accumulation of autofluorescent material, measured by the average area of the autofluorescent compartments and the average number of autofluorescent objects, compared to the control cells (Figure 4A–D). Increased autofluorescence was present in CLN5Y392X iPSC-derived cells in the all four cell densities analysed in the study, and was statistically significant at the highest cell densities (Figure 4C,D). Analyses of subunit C by a specific antibody staining also showed an accumulation of the protein in the CLN5-deficient cells (Figure 4E,F) that was further confirmed by high-content image analysis (Figure 4G–I).

### 2.3. CLN5Y392X iPSC-Derived Neural Lineage Cells Show Changes in Sphingolipid Transport

**Figure 5:**
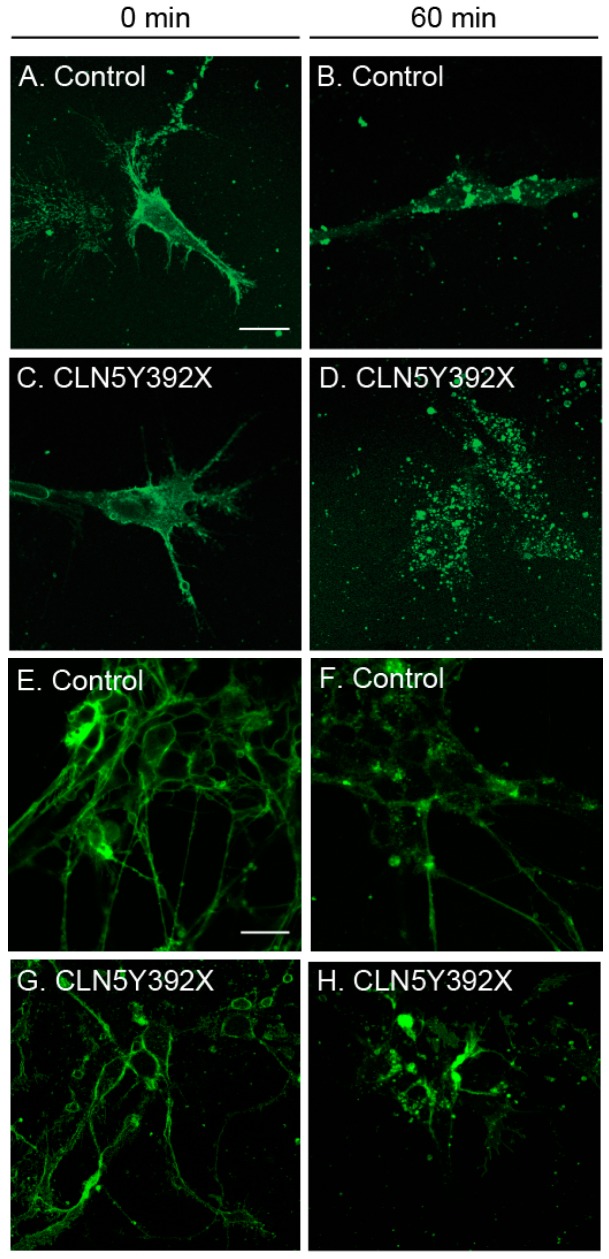
Altered sphingolipid transport in CLN5Y392X iPSC-derived neural lineage cells. Control and CLN5Y392X cells were fed with a fluorescent glycosphingolipid analogue BODIPY-Lactosylceramide (BODIPY-LacCer). Immediately after feeding (0 min chase), BODIPY-LacCer was located at the plasma membrane (**A**,**C**,**E**,**G**); After chasing for 60 min, BODIPY LacCer displayed a typical Golgi distribution in the control cells (**B**,**F**); whereas the labelling was more dispersed in CLN5Y392X iPSC-derived neural lineage cells (**D**,**H**). Images shown in the figure are representative of experiments performed on neural lineage cells from two individual control and two CLN5-deficient hiPSC lines in two separate experiments (two different maturation time points, (**A**–**D**,**E**–**H**)). Bar 20 µm.

We previously showed that sphingolipid transport is altered in *Cln5* ko mouse macrophages [15]. Therefore, we analysed whether we could see the same alterations in neural lineage cells derived from CLN5 patient iPSCs. The fluorescent glycosphingolipid analogue BODIPY-Lactosylceramide (LacCer) is normally transported from the plasma membrane to the Golgi compartment, but is sequestered in the lysosomes in a number of lysosomal storage diseases and is used as a general screen for sphingolipid storage diseases [36]. The CLN5Y392X iPSC-derived neural lineage cells were labelled with the BODIPY-LacCer after which the intracellular trafficking of the fluorescent lipid was followed. At 0 min chase, both control and CLN5Y392X iPSC-derived neural lineage cells displayed prominent BODIPY-LacCer labelling at the plasma membrane (Figure 5A,C). At 60 min chase, control cells displayed punctate BODIPY-LacCer staining in the perinuclear area, which is indicative of BODIPY-LacCer reaching the Golgi (Figure 5B). In CLN5Y392X iPSC-derived cells, the staining was more dispersed, indicating clear alterations in BODIPY-LacCer trafficking (Figure 5D). These results were also repeated in more mature cultures (Figure 5E–H). After 7 days in an adherent culture, control cells showed comparable staining pattern (Figure 5F) to that detected on day 5, while CLN5-deficient neural lineage cells were abnormally stained by BODIPY-LacCer until day 10 (Figure 5H). These results suggest that similar to our previous findings in the *Cln5* ko mouse model, sphingolipid transport from endo-lysosomes to the Golgi is also disturbed in CLN5Y392X iPSC-derived neural lineage cells.

## 3. Discussion

In this study we describe the generation and characterisation of human induced pluripotent stem cells derived from a CLN5 patient, suffering from a variant form of pediatric neurodegenerative NCL. The generated cell lines harbour the *CLN5* c.1175_1176delAT (p.Tyr392X) mutation, and thus, represent the most common cause of CLN5 disease [1,5,37].

The function of the lysosomal CLN5 protein, as well as the cellular consequences of *CLN5* mutations, is still largely unknown. So far, the *Cln5* ko mouse model has been the most valuable tool to study the pathogenesis of the disease, although it does not fully recapitulate the disease phenotype in humans [14,15,16]. Lack of characteristic seizures in the mouse model suggest that the human genome may contain regulatory or modifying elements, which may not be present or that important in the mouse. Interestingly, the phenotypes of some CLN5 patients have been suggested to be influenced by mutations in other genes playing a modifying role in CLN5 disease [38,39]. Further, *CLN5* mutations have recurrently been found among patients suffering from other forms of NCLs, suggesting a modifying role for the CLN5 protein itself (NCL mutation database, http://www.ucl.ac.uk/ncl, and references therein). Patient-derived iPS cells include not only the mutated *CLN5*, but also contain all the genetic factors that may play an important role in the pathology of CLN5 disease in humans. In this study, we show that CLN5Y392X iPSC-derived neural lineage cells of human origin represent typical hallmarks of CLN5 disease, the accumulation of autofluorescent storage material and subunit C of mitochondrial ATP synthase and thus, provide a promising model to study the characteristic features of the human disease.

In addition, we detected morphological changes in the intracellular organelles of the CLN5Y392X iPSC-derived neural lineage cells. Both lysosomal compartment and the ER were clearly enlarged in the CLN5Y392X iPSC-derived cells compared to the control cells. Accordingly, it was just recently reported that LAMP-1-positive lysosomal compartments of fetal neuronal cultures prepared from CLN5-deficient sheep have similar appearance as observed in this study in patient-specific neural lineage cells [40]. Increased lysosomal size is a typical phenomenon for lysosomal disorders, which accumulate storage material in the lysosomes. Disturbances in the lysosomal structures have also previously been seen in neuronal cells derived from CLN3 iPSC, and interestingly, these abnormalities are suggested to occur prior to the lysosomal storage [41]. Therefore, it is possible that NCL proteins also participate in other functions of lysosomes than degradation and transport across the lysosomal membrane. Interestingly, CLN5 and CLN3 have been suggested to share a common interaction partner, Rab7, a regulator of endosomal-lysosomal membrane trafficking [29,30]. However, further studies are needed to clarify the role of these NCL proteins with respect to Rab7 in order to understand their exact role in the endosomal/lysosomal system.

Based on our results and the data of others [41], it seems that the enlargement of the ER is a recurrent phenomenon in neural lineage cells differentiated from NCL patient-derived iPSCs. Increased ER size is often an indicator of ER stress, which has been associated with other NCL disorders [42,43,44], and together with oxidative stress, is suggested to be at least partially responsible for neuronal death detected in NCLs [45]. ER stress may well occur also in CLN5-deficient cells since most of the disease mutations lead to the retention of the protein to the ER [17]. On the other hand, recombinant CLN5 has been reported to interact with the ER-resident NCL proteins, CLN6 and CLN8 [24], and with the ER quality control machinery [21], suggesting that CLN5 may have an extralysosomal role along the trafficking pathway from the ER to the lysosomes [17,18]. Therefore, abnormal ER in CLN5 deficiency may well result directly from the lack of functional CLN5.

Most importantly, we observed that CLN5Y392X iPSC-derived neural lineage cells have defective sphingolipid transport, previously shown to occur in macrophages isolated from the *Cln5* ko mouse [15]. This finding further verifies that disturbances in sphingolipid metabolism are recurrently associated with CLN5 deficiency. The importance of sphingolipid metabolism in CLN5 pathogenesis is also supported by the previous findings from the *Cln5* ko mouse that hypomyelination occurs early and precedes neuronal loss in the thalamocortical system [15]. In fact, abnormalities both in sphingolipid and cholesterol transport and metabolism seem to be a common theme among NCLs (reviewed in [46]), and should be the focus of future studies.

Patient-derived iPS cells and their differentiated derivatives may well provide better models for NCL disorders. We showed here that CLN5Y392X iPSC-derived neural lineage cells express typical characteristics of the CLN5 disease and replicate functional defects detected in the *Cln5* mouse model. iPSC technology also allows investigation of human neurons during development, thus providing studies of disease-specific pathways prior to and during disease onset. Therefore, studies of disease-relevant human cells may lead to better understanding of the function of CLN5 protein and pathology of the disease. More importantly, iPS cells and their derivatives may be used as platforms for performing high-throughput screenings of large chemical libraries to identify novel drug candidates for the disease.

## 4. Materials and Methods

### 4.1. Ethical Issues

The study was conducted in accordance with the Declaration of Helsinki. The work with patient dermal fibroblasts in this study has been approved by the ethical permission no. 116/13/03/00/2012 and HUS/2235/2016 (13 December 2016), provided by the ethical committee of the hospital district of Helsinki and Uusimaa, Finland.

### 4.2. Cell Lines

Fibroblasts were cultured in Dulbecco’s modified Eagle’s medium (DMEM) supplemented with 20% fetal bovine serum (FBS), l-glutamine, and antibiotics. The characterised control iPSC lines from healthy individuals have been described earlier [33,34], and were used in this study by permission, no. 17/13/03/00/2011/ agreement 2014-31a.

### 4.3. Generation and Characterisation of CLN5Y392X iPSC Lines

Fibroblasts from a CLN5 male patient carrying the most common *CLN5* mutation (c.1175_1176delAT, p.Tyr392X) were reprogrammed by using a Sendai virus-mediated delivery of the four Yamanaka factors (CytoTune-iPS Reprogramming Kit, Life Technologies, Carlsbad, CA, USA). Emerging iPSC colonies (CLN5Y392X iPSCs) were grown on mitomycin C-inactivated SNL-feeder cells [47], kindly provided by the Wellcome Trust Sanger Institute (Cambridge, UK). Colonies were selected on the basis of ESC-like colony morphology, and further expanded on feeders in hES medium (DMEM/F12 supplemented with GlutaMAX, 20% KnockOut-Serum Replacement, 0.1 mM β-mercaptoethanol, 1× nonessential amino acids; all from Life Technologies) containing 6 ng/mL recombinant human fibroblast growth factor-basic (FGF-basic) (PeproTech, Rocky Hill, NJ, USA) and antibiotics, or on Matrigel-coated (BD Biosciences, Erembodegen, Belgium) cell culture plates in Essential 8 medium (E8, Life Technologies) containing antibiotics.

Expression of pluripotency-associated markers, TRA-1-60, OCT3/4 and SSEA3 was confirmed by immunofluorescence microscopy (Zeiss Axioplan 2, Jena, Germany) and colorimetric assay, respectively, according to the instructions of the manufacturer (ES Cell Characterisation kit, Merck Millipore/Merck, Darmstadt, Germany). Expression of other ES cell marker genes (*NANOG*, *OCT3/4*, *REX1*, and *TDGF1*) in selected iPSC clones was verified by RT-PCR. Primer sequences are described by Vuoristo et al. [48] (*OCT3/4* and *NANOG*) and Trokovic et al. [49] (*TDGF1* and *REX1*).

The clearance of the Sendai virus and the virus-delivered transgenes was confirmed by RT-PCR using primers recommended by the manufacturer (CytoTune-iPS Reprogramming Kit, Life Technologies).

The presence of the CLN5 mutation in each established CLN5Y392X iPSC line was verified by sequencing.

### 4.4. Pluripotency of CLN5Y392X iPS Cells

To study the spontaneous differentiation potential of the produced CLN5Y392X iPSC clones, embryoid bodies (EBs) were grown on ultra-low attachment plates (Corning, New York, NY, USA) in hES medium without FGF-basic for 14 days, and then dissociated and plated on gelatin-coated tissue culture plates. Differentiated cells were fixed with 4% paraformaldehyde (PFA) and stained with rabbit anti-α-fetoprotein (AFP) (1:500, Dako/Agilent Technologies, Santa Clara, CA, USA), mouse anti-vimentin (1:2000, Dako), or rabbit anti-βIII-tubulin (1:1000, Covance, Princeton, NJ, USA) antibodies for indication of endoderm, mesoderm and ectoderm, respectively. In each experiment, the cell nuclei were stained with Hoechst 33258 (Molecular Probes, Eugene, OR, USA), and each primary antibody labelling was detected by an appropriate fluorophore-conjugated secondary antibody (Jackson ImmunoResearch Laboratories, West Grove, PA, USA). The stainings were viewed using a Zeiss Axioplan 2 microscope.

### 4.5. Differentiation of iPS Cells towards Neural Lineage Cells

Neural differentiation of iPSCs was performed according to previously described protocols [50,51]. Briefly, iPSC colonies grown on feeders were first monitored by light microscopy followed by scraping the feeders from the plate using a pipette tip or a needle. The remaining iPSC colonies were gently detached with collagenase IV (Life Technologies) and plated on ultra-low attachment plates (Corning) in neuronal induction medium, NIM (DMEM/F12:Neurobasal medium, 1:1, supplemented with 1× GlutaMAX, 1× B27 *w*/*o* vitamin A, 1× N2, and antibiotics, all from Life Technologies or PeproTech). NIM was changed to neuronal proliferation medium, NPM (NIM supplemented with 25 ng/mL recombinant human FGF-basic), after 48 h. The formed neurospheres were manually cut into smaller pieces once a week, and media were changed every three days for a minimum of five weeks. For further maturation, neurospheres were cut mechanically into small aggregates and then gently dissociated with TrypLE Select (Life Technologies), filtered through 40 µm cell strainer, and plated at different densities on poly-l-ornithine (15 µg/mL, Sigma-Aldrich, Saint Louis, MO, USA) and laminin (10 µg/mL, Sigma-Aldrich) coated plates in NIM. The differentiation was followed daily by light microscopy.

### 4.6. Immunofluorescence Analyses of iPSC-Derived Neural Lineage Cells

iPSC-derived neural lineage cells differentiated for 7–14 days in NIM were fixed with 4% PFA for immunofluorescence analyses. Mouse anti-MAP2 (1:200, Sigma-Aldrich) antibody was used to detect neurons. Lysosomes were stained by a mouse anti-LAMP-1 antibody (1:100, H4A3 from the Developmental Studies Hybridoma Bank, DSHB, Iowa city, IA, USA), and endoplasmic reticulum (ER) was stained by a mouse anti-PDI antibody (1:200, BD Biosciences). Subunit C was stained with a rabbit antibody against subunit C of mitochondrial ATP synthase, a generous gift from David Palmer (Lincoln University, Lincoln, New Zealand) [52]. Where indicated, the cell nuclei were stained with Hoechst 33258 (Life Technologies). Secondary antibodies were obtained from Jackson ImmunoResearch Laboratories.

The stainings were viewed with a Zeiss Axioplan 2 fluorescence microscope and, for quantitative image analysis, with a ScanR a high-content epifluorescence microscope (Olympus, Hamburg, Germany), followed by image analysis as described below.

### 4.7. Quantitative Image Analyses

ScanR (Olympus), a modular epifluorescence microscope designed for fully automated high-content image acquisition was utilised. All experiments were performed in replicates, and for each replicate, several different fields of view were analysed using ScanR Analysis software 1.3.0.3.2.3 (Olympus). The number of replicates and the total amount of cells counted for each analysis are indicated in the respective figure legends. Results are shown as the mean of the values of individual replicates with each value representing the whole pool of cells within several fields of view. Therefore, values obtained from each individual replicate were normalised to analysed cell count (Hoechst positive compartments) in each replicate.

All images were manually controlled for quality and for potential errors, and an automated background correction and thresholding was applied to each channel. The cell nuclei stained with Hoechst 33258 were defined and counted as main objects for each experiment. Other compartments to be analysed were defined as subobjects.

LAMP-1-positive lysosomal compartments were analysed for their total size, number and intensity. The size of the ER was determined by measuring the area of PDI-stained structures. In addition, the intensity of PDI staining was calculated. Autofluorescence and subunit C-stained compartments were analysed by measuring the total area (autofluorescence only) and the number of autofluorescent (green)/subunit C-stained (red) objects.

Student’s *t* test was used for statistical analyses. Image processing was performed with Adobe Photoshop CS4 and Adobe Illustrator CS4 (Adobe Systems Inc., San Jose, CA, USA).

### 4.8. Analyses of Lipid Transport in iPSC-Derived Neural Lineage Cells

Fluorescent sphingolipid transport was analysed essentially as described previously [36]. Briefly, iPSC-derived neural lineage cells were washed four times with ice cold Eagle’s minimal essential medium (EMEM, Life Technologies) and incubated for 30 min on ice with EMEM supplemented with 5 μM BODIPY FL C5-lactosylceramide (Life Technologies). The cells were then washed four times with ice-cold EMEM and plasma membrane labelling was assessed using fluorescence microscopy. The label was chased into the cells by incubation in EMEM at +37 °C for 1 h, after which the cells were transferred onto ice and washed 6 times, for 10 min, with EMEM containing 5% bovine serum albumin (BSA) to remove the non-internalised label from the plasma membrane. The medium was changed to cold EMEM, and the BODIPY FL C5-lactosylceramide internalisation was assessed using an Olympus IX71 fluorescence microscope with a UAPO 40×/NA1.35 oil objective and 500 nm excitation and 520 nm emission filters.

## Supplementary Materials

Supplementary materials can be found at www.mdpi.com/1422-0067/18/5/955/s1.

## Abbreviations

NCL	Neuronal ceroid lipofuscinoses
CLN	Ceroid lipofuscinosis neuronal
iPSC	Induced pluripotent stem cell
hESC	Human embryonic stem cell
NPC	Neural progenitor cell
EB	Embryoid body
