# Supplementary material for: Induced Pluripotent Stem Cells Derived from a CLN5 Patient Manifest Phenotypic Characteristics of Neuronal Ceroid Lipofuscinoses
Source: Int J Mol Sci. 2017 May 3;18(5):955. doi: 10.3390/ijms18050955 (PMC5454868; doi:10.3390/ijms18050955)
Supplement: Supplementary file 1 [file ijms-18-00955-s001.pdf]

Induced pluripotent stem cells derived from a CLN5 patient manifest phenotypic characteristics of the neuronal ceroid lipofuscinoses

Kristiina Uusi-Rauva, Tea Blom, and Carina von Schantz-Fant, Tomas Blom, Anu Jalanko and Aija Kyttälä

Supplementary Materials and Methods

Analysis of TGN and CI-MPR in Control and CLN5 patient's Fibroblasts

For the analyses of the steady-state localisation of TGN in control and CLN5 patient fibroblasts, the cells were grown on coverslips, fixed and stained with a sheep anti-TGN46 antibody (1:1000, Serotec). To detect possible steady-state mislocalisation of the CI-MPR, fibroblasts were fed for 2h with 0.2 µM LysoTracker Red (DND-99, Molecular Probes) to stain the lysosomes, then fixed and stained with a goat anti-CI-MPR (1:200, a kind gift from Professor Stefan Höning, University of Köln, Germany). To further examine a possible degradation of CI-MPR in CLN5-deficient fibroblasts, the cells were treated for 4 h with 50 µg/ml cycloheximide (Sigma-Aldrich) to block the protein synthesis, which has previously been used to identify lysosomal mistargeting of CI-MPR [35]. The cells were then fixed and stained for CI-MPR as above.

The cell nuclei were stained with Hoechst 33258 (Life Technologies), and each primary antibody labelling was detected by an appropriate fluorophore-conjugated secondary antibody (Jackson ImmunoResearch Laboratories). Each staining was viewed with a Zeiss Axioplan 2 microscope and for quantitative image analysis, with a ScanR, a high-content epifluorescence microscope (Olympus), combined with image analysis. TGN46 and CI-MPR-positive compartments were analysed by measuring the total area and perimeter of the stained structures. Student's t test was used for statistical analyses. Image processing was performed with Adobe Photoshop CS4 and Adobe Illustrator CS4 (Adobe Systems Inc.).

Analysis of TGN46 and CI-MPR in CLN5-depleted fibroblasts

Appropriate control siRNAs, AllStars Cell Death Control siRNA (transfection control, Qiagen) and Ambion Negative Control #1 siRNA (scrambled, Ambion), and three different siRNAs for CLN5 (Ambion Silencer® Select Human siRNA Library V4), each with a final concentration of 12 nM per well, were separately transferred to replicate wells of a black clear bottom tissue culture treated 384-well plate (Corning) using an acoustic droplet ejection method with an Echo 550 liquid handler (Labcyte) [53]. Prior to transfection, 5 µl of Opti-MEM medium (Gibco) containing 75 nl of Lipofectamine RNAiMAX (Invitrogen) was added per well and the plate was mixed for 15 min. After mixing, 750 cells per well were added in 20 µl of fibroblast culture medium with antibiotics. Transfected cells were incubated at 37°C for 4 days in a cell incubator (HERACell 240, Thermo Scientific) in an atmosphere of 5 % CO_2. The siRNA-transfected and mock-treated fibroblasts were then fixed and stained for nuclei and TGN46 or CI-MPR as described in materials and methods. Samples were then analysed by automated high-content immunofluorescence microscopy followed by quantitative image analysis. TGN46 and CI-MPR-positive compartments were analysed by measuring perimeter and area of the stained structures as described in materials and methods. In addition, the distribution of TGN46-positive compartments within the cells was measured by first creating a mask over the nucleus and a circular area just outside the nucleus (representing the area where the Golgi normally resides, area 1). Another mask was created outside the first mask (covering the rest of the stained areas, area 2). Representative images of created masks are shown in Supplemental figure 5. The ratio of the amount, measured as the area, of TGN46-stained objects between these two masks was calculated.

Karyotyping

For karyotype analysis, iPSCs cultured on Matrigel were detached with EDTA and pelleted for DNA extraction by E.Z.N.A. Tissue DNA extraction kit (Omega Bio-Tek) according to the manufacturer´s instructions. Chromosomal integrity was analysed at the Finnish Microarray and Sequencing Centre (FMSC, Turku, Finland) by a KaryoLite™ BACs-on-Beads™ method (KaryoLite™ BoBs™, Perkin Elmer) [48,54] with two technical replicates per sample.

Supplementary Figures

Supplementary figure 1. Characterisation of CLN5Y392X iPSCs

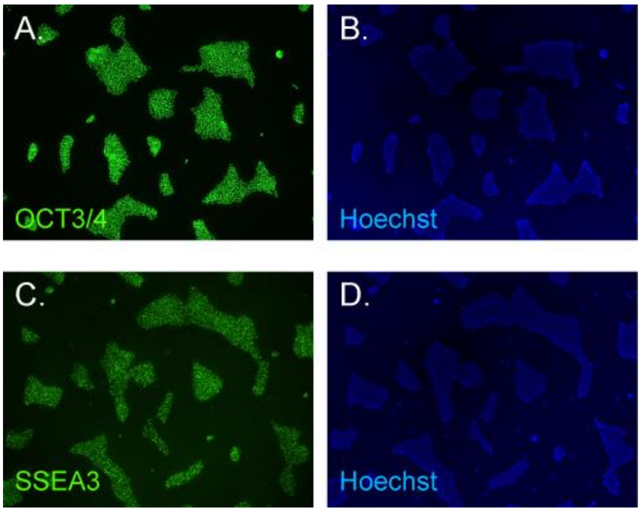

CLN5Y392X iPSC colonies were stained for stem cell markers OCT3/4 and SSEA3 (**A** and **C**, respectively, shown for one representative clone). Respective nuclear stainings shown on the right (**B** and **D**).

Supplementary figure 2. Karyotype analysis of the CLN5 patient iPS cell lines.

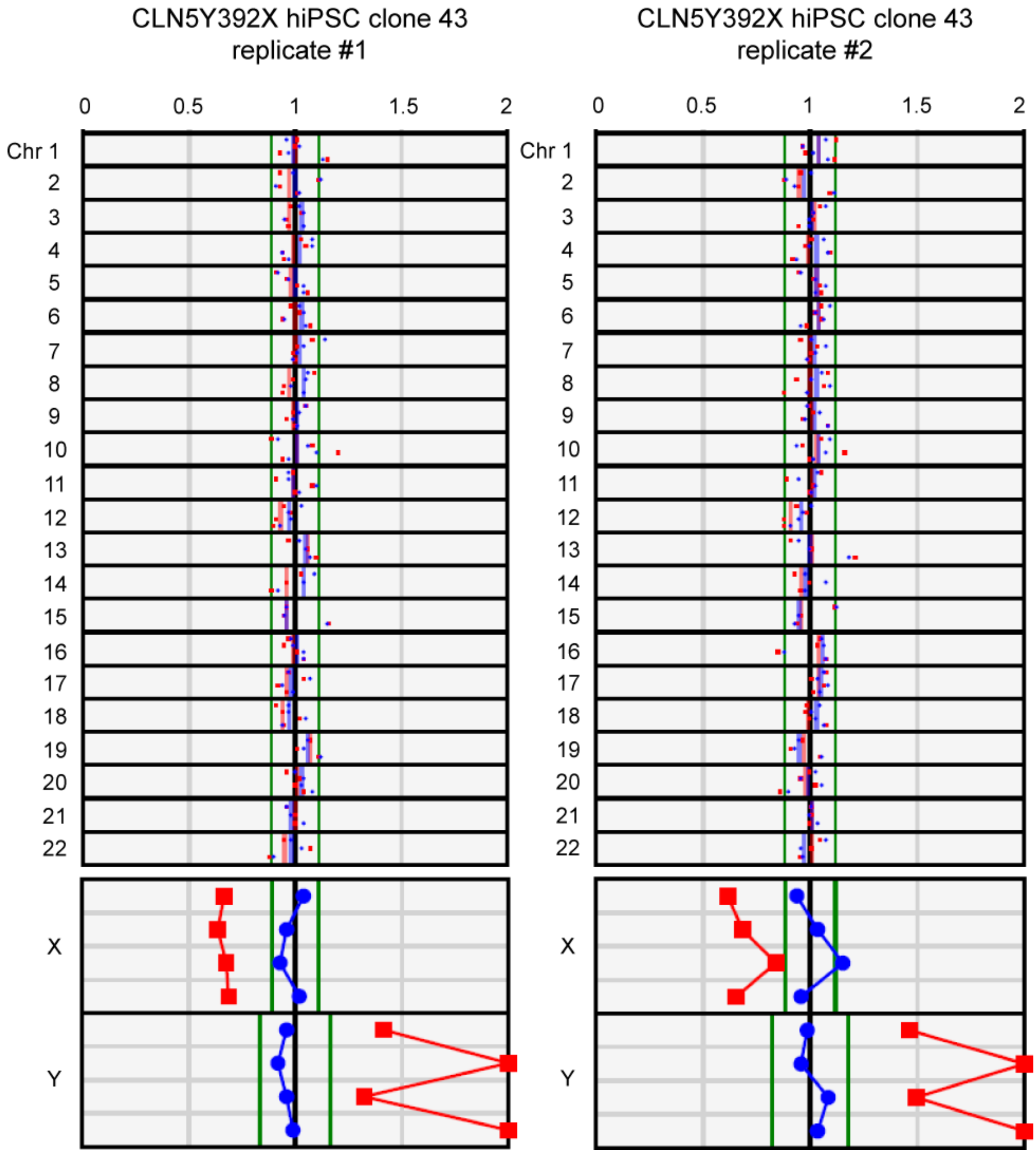

The chromosomal integrity of established CLN5 patient iPS cell lines was analysed by KaryoLite™ BoBs™ (Perkin Elmer). The data of both analysed technical replicates for one representative clone shown only. The blue and red dots indicate the normalised chromosomal signal ratios of CLN5Y392X iPSC DNA against the male (blue) and female (red) reference DNAs with normal genotype. Each chromosome (Chr) was analysed with two probe sets per arm (targeted to proximal and distal regions of the arm), except for the acrocentric chromosomes 13-15, 21 and 22, where the one existing arm is covered with three probe sets. In case of chromosomal abnormalities, both signal ratios should clearly exceed the calculated threshold values in both replicates, whereas in the case of normal chromosomes, the signal ratios should reside inside the reference area around value 1. Based on the analysis of two technical replicates each analysed CLN5Y392X iPS cell line was confirmed to represent a male patient and no major chromosomal aberrations were found in any of the analysed clones.

Supplementary figure 3. Analysis of TGN in control and CLN5 patient's fibroblasts

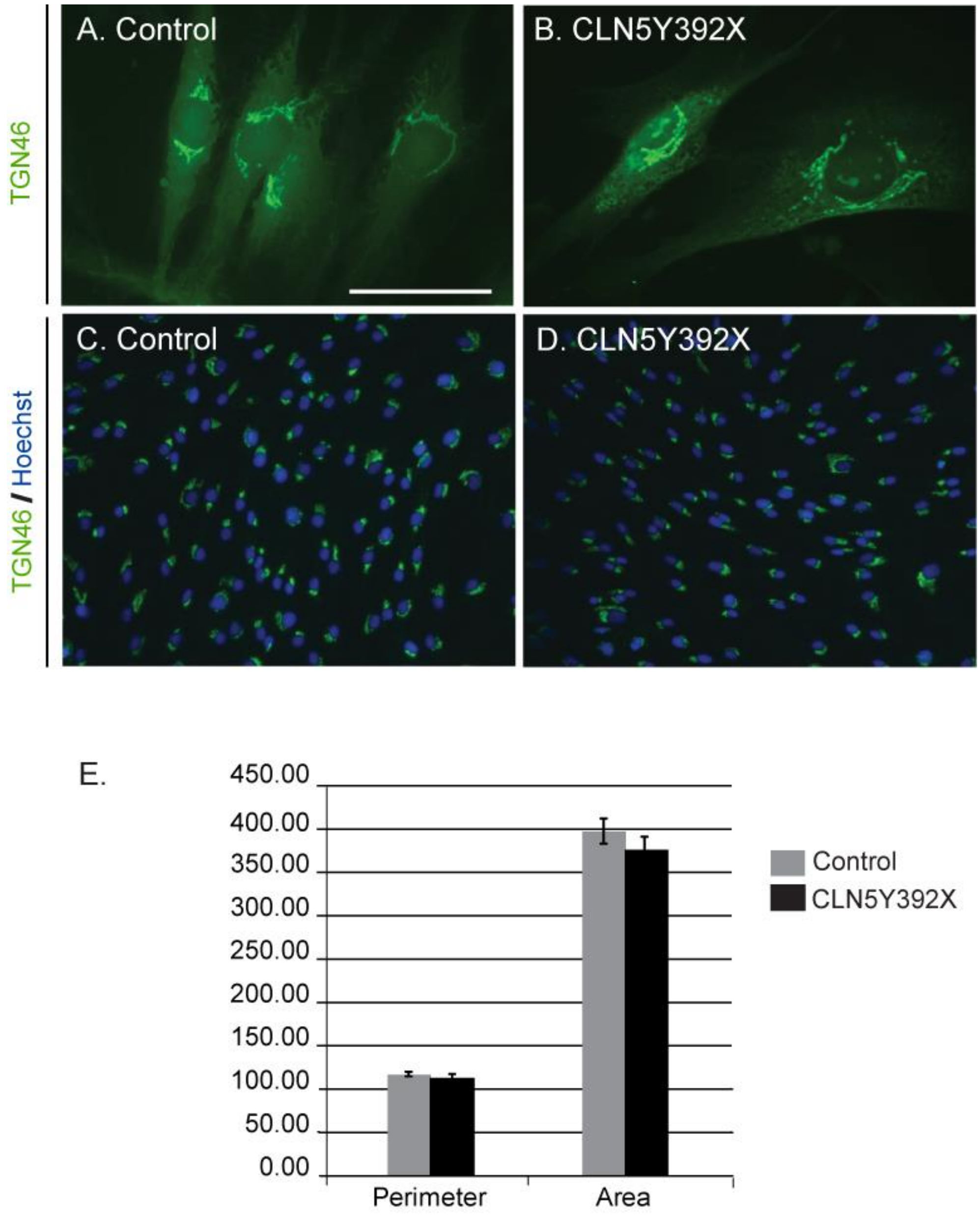

TGN in control and CLN5 patient fibroblasts. Steady-state localisation of the trans-Golgi network (TGN, stained by an anti-TGN46 antibody) in control (**A**) and in CLN5 patient (CLN5Y392X) (**B**) fibroblasts viewed by traditional epifluorescence microscope. Bar (A, B) 50 µm. Automated high-content screening microscopy followed by image analysis of the TGN46 (green) and nuclear (blue) staining in human fibroblasts (**C-E**). Representative microscopy images are shown for the control (**C**) and CLN5 patient (**D**) cells. Quantitative image analysis shows the average values (arbitrary units) of total size, measured as perimeter and area, of the TGN46-positive structures in the control and CLN5-deficient fibroblasts (**E**). All columns represent the average values of 3-4 replicates normalised to the amount of cells analysed in the replicate (the average of 350 cells per replicate). Error bars represent standard deviations (stdevp).

Supplementary figure 4. Analysis of CI-MPR in control and CLN5 patient fibroblasts

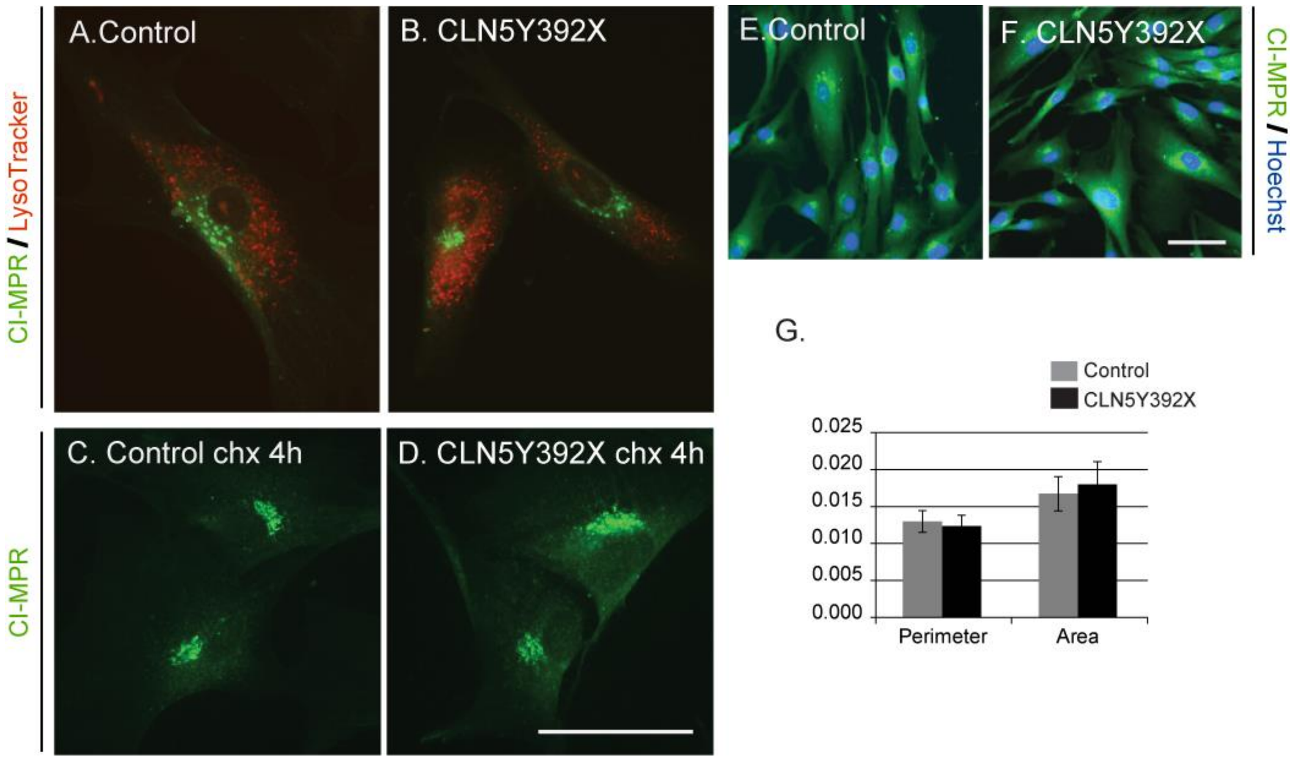

CI-MPR in control and CLN5 patient fibroblasts. Cation-independent mannose 6-phosphate receptor (CI-MPR) staining in human fibroblasts viewed by traditional epifluorescence microscope (**A-D**). Steady-state localisation of CI-MPR (green) and lysosomes (LysoTracker, red) in control (**A**) and in CLN5 patient (CLN5Y392X) (**B**) fibroblasts. Localisation of CI-MPR after blocking the protein synthesis with cyclohexamide (chx) for 4h (**C, D**). Both control (**C**) and CLN5 (**D**) fibroblasts show compact CI-MPR staining with no apparent loss of the protein. Bar (A-D) 50 µm. Automated high-content microscopy followed by image analysis was performed for CI-MPR (green) and nucleus (blue) in human fibroblasts in steady-state conditions (**E-G**). Representative images are shown for control (**E**) and CLN5 patient (**F**) cells. Bar (E, F) 50 µm. Quantitative image analysis shows the average size, measured as perimeter and area (arbitrary units), of the CI-MPR-positive compartments in control and CLN5-deficient fibroblasts (**G**). All columns represent the average values of three replicates corrected for the amount of cells analysed per replicate (average of 1050 cells per replicate). Error bars represent standard deviations (stdev).

Supplementary figure 5. TGN in control and CLN5-depleted human fibroblasts.

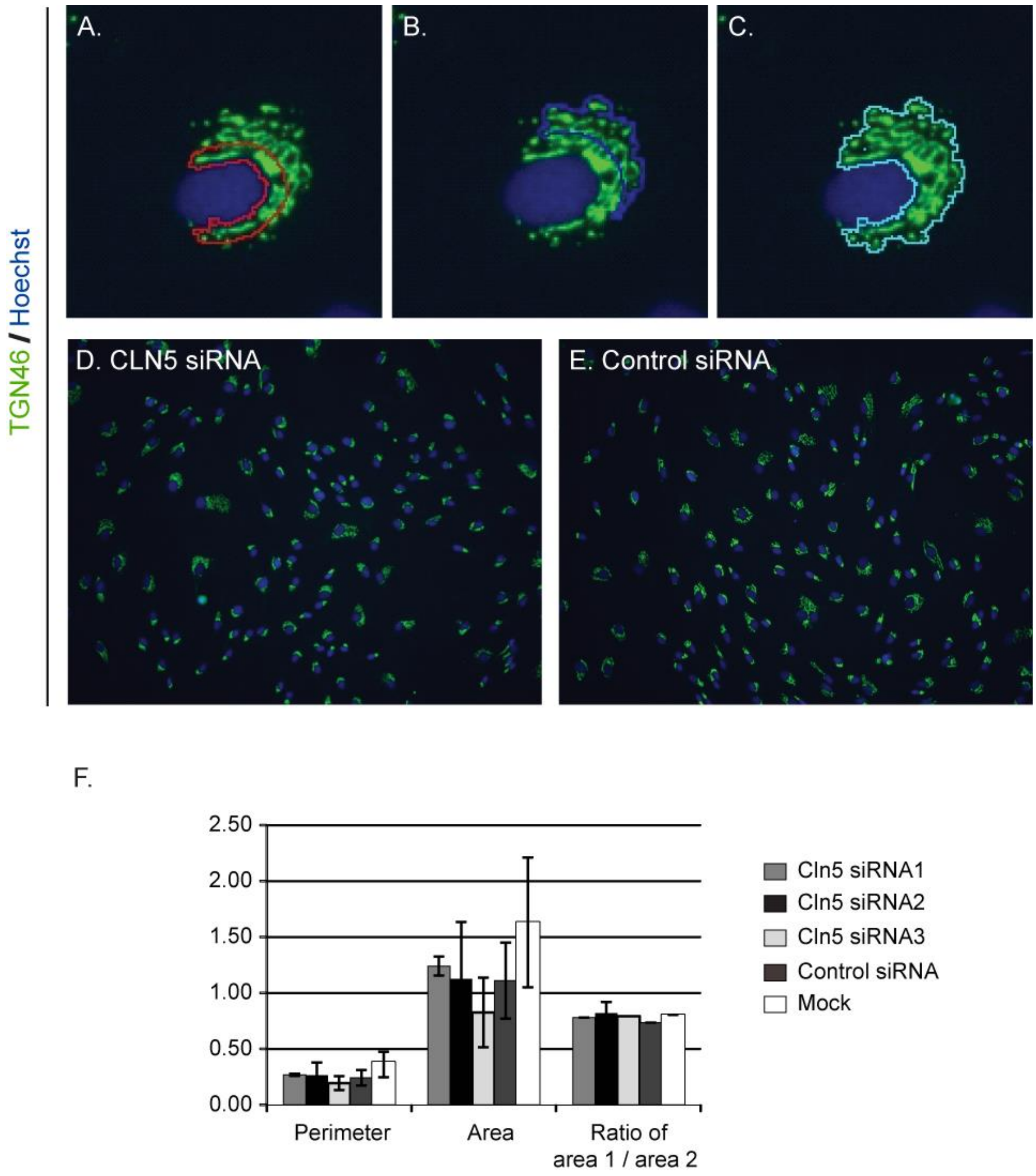

Automated high-content microscopy followed by image analysis was performed for control siRNA (scrambled), CLN5 siRNA and mock-treated human fibroblasts stained for TGN (TGN46, green) and nuclei (Hoechst 33258, blue). The marked areas (**A**-**C**) visualise the masks used to analyse the TGN46 distribution in the cells. Area 1 (red), the area closest to the nucleus (**A**). Area 2 (blue), the area further away from the nucleus (**B**). Total area of staining (light blue) (**C**). Representative microscopy images of TGN46-stained human fibroblasts transfected with representative siRNA for CLN5 (**D**) and of cells transfected with control siRNA (**E**). The data from the quantitative image analysis of fibroblasts mock-treated or transfected with control (scrambled) or CLN5 siRNAs (**F**). The average values (arbitrary units) of size, measured as perimeter and area, as well as of the distribution measured as the ratio of the two indicated areas containing TGN46-positive compartments. Columns represent the average values of two replicates in each condition. Error bars represent standard deviations (stdev).

Supplementary figure 6. CI-MPR in control and CLN5-depleted human fibroblasts.

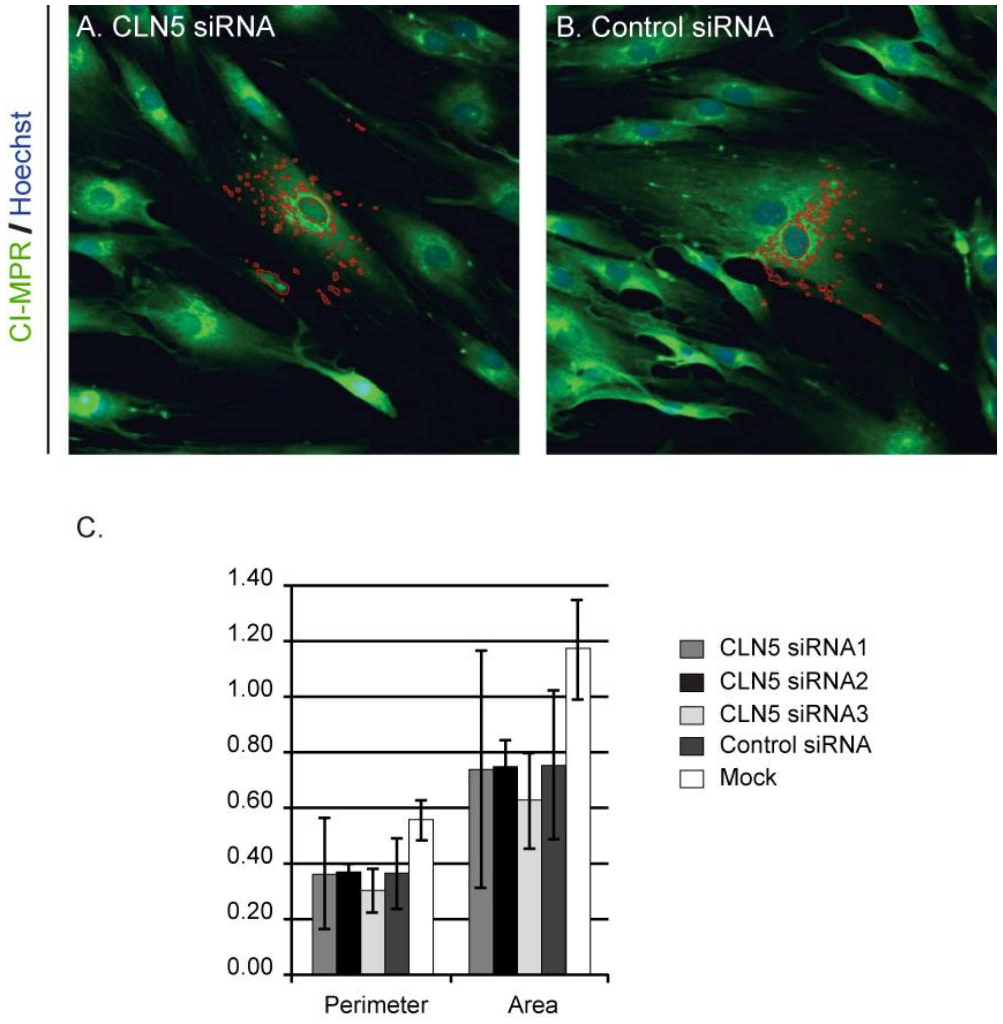

Automated high-content microscopy followed by image analysis of control siRNA, CLN5 siRNA and mock-treated human fibroblasts stained for CI-MPR (green) and nuclei (Hoechst 33258, blue). Microscopy images of cells transfected with representative siRNA for CLN5 (**A**) and of cells transfected with control siRNAs (**B**). The red area in both pictures visualises the mask used for analysing the CI-MPR staining. Quantitative image analysis shows the average values (arbitrary units) of size, measured as perimeter and area, of the CI-MPR-positive compartments (within the created mask) in each condition (**C**). Error bars represent standard deviations (stdev).

Supplementary References

53. Von Schantz, C.; Saarela, J. High throughput sirna screening using reverse transfection. *Methods Mol. Biol.* **2016**, *1470*, 25–37.
54. Lund, R.J.; Nikula, T.; Rahkonen, N.; Narva, E.; Baker, D.; Harrison, N.; Andrews, P.; Otonkoski, T.; Lahesmaa, R. High-throughput karyotyping of human pluripotent stem cells. *Stem Cell Res.* **2012**, *9*, 192–195.
